# Adipose-Derived Stromal Cell Therapy Affects Lung Inflammation and Tracheal Responsiveness in Guinea Pig Model of COPD

**DOI:** 10.1371/journal.pone.0108974

**Published:** 2014-10-20

**Authors:** Azadeh Feizpour, Mohammad Hossein Boskabady, Ahmad Ghorbani

**Affiliations:** 1 Neurogenic inflammation Research Centre and Department of Physiology, School of Medicine, Mashhad University of Medical Sciences, Mashhad, Iran; 2 Pharmacological Research Center of Medicinal Plants, School of Medicine, Mashhad University of Medical Sciences, Mashhad, Iran; University of Pittsburgh, United States of America

## Abstract

The effects of adipose derived stromal cells (ASCs) were evaluated on tracheal responsiveness and biochemical parameters in guinea pigs model of chronic obstructive pulmonary disease (COPD). Thirty six guinea pigs were divided into 6 groups including: Control, COPD, COPD+intratracheal delivery of PBS (COPD+ITPBS), COPD+intravenous delivery of PBS (COPD+IVPBS), COPD+intratracheal delivery of ASCs (COPD+ITASC) and COPD+intravenous injection of ASCs (COPD+IVASC). COPD was induced by exposing animals to cigarette smoke for 3 months. Cell therapy was then performed and after 14 days, tracheal responsiveness, concentration of interleukin-8 (IL-8) in serum and broncho-alveolar lavage fluid (BALF), as well as total and differential white blood cells (WBC) counts were evaluated. Tracheal responsiveness, total WBC counts, neutrophil and eosinophil percentage in BALF as well as concentration of IL-8 in serum and BALF significantly increased but lymphocyte percentage decreased in COPD compared to the control group (*P*<0.05 to p<0.001). Cell therapy was able to restore the tracheal hyper-responsiveness and the increased IL-8 concentration in serum and BALF of COPD-ITASC but not COPD-IVASC animals (*P*<0.05 for all cases). Total WBC in BALF also showed a significant decrease in both treated groups and the percentages of eosinophils, neutrophils and lymphocytes in BALF were reversed in COPD-ITASC compared to COPD-ITPBS animals (*P*<0.05 to *P*<0.001). Therefore, intratracheal cell therapy with ASC can decrease tracheal hyperresponsiveness and lung inflammation in cigarette smoke induced-COPD which may be helpful in attenuation of the severity of disease in patients suffering from COPD.

## Introduction

Chronic obstructive pulmonary disease (COPD) is expanding as the fourth leading cause of death in the world [1]. Chronic bronchitis, chronic obstructive bronchiolitis, increased airway responsiveness and emphysematous destruction of the lung parenchyma are the most usual pathologic changes observed in this progressive lung disease [2]–[4]. These changes are mostly due to repetitive exposure to cigarette smoke which induces inflammatory responses, oxidative stress and protease-antiprotease imbalance in the lung [4]–[6].

The current treatments for COPD are not able to restore airflow limitation and accelerated loss of lung function [7]. Adipose derived stromal cells (ASCs) and mesenchymal stem cells (MSCs) derived from ASCs are reported to have some beneficial action on pulmonary diseases [8]–[11]. The ASCs ameliorate lung injury through secretion of different factors and their paracrine effects [8]. The cells produce a large amount of hepatocyte growth factor and increase alveolar and vascular regeneration in the lung [9]. Intratracheal administration of MSCs reduces the destruction in elastase-induced emphysema through secretion of paracrine factors such as epidermal growth factor [10]. Also, it has been reported that MSCs interfere with inflammation responses and exert immune modulatory effects [11]–[13]. For example, Gupta et al., showed that beneficial effect of bone marrow-derived MSCs on endotoxin-induced lung injury is independent of their ability to engraft in the lung and is related to down-regulation of proinflammatory responses to endotoxin (e.g. reducing TNF-alpha in the bronchoalveolar lavage and plasma) and increasing anti-inflammatory cytokines (e.g. IL-10) [12].

As far as our knowledge, the potential therapeutic effect of ASCs hasn’t been previously studied in cigarette induced COPD in guinea pigs, the main model of respiratory diseases. Therefore, the present study aimed to elucidate the effects of local and systemic injection of ASCs on tracheal responsiveness and pulmonary inflammation in COPD animals. The ASCs have the advantage of being easily accessible and having lower ethical context in comparison with other cell therapy sources such as bone marrow stem cell.

## Materials and Methods

### Animal exposure to cigarette smoke

Exposure of guinea pigs to cigarette smoke was performed according to the previously described method [14]. Briefly, the animals were placed in a two compartment box: one held the body of the animal and the other one held its head (dimensions: 15×12×7 cm). Twenty millilitre puffs of cigarette smoke were drawn using a syringe and exhausted into the animals’ head chamber (2 puffs/min). Exposure of animals to each cigarette lasted for 8–9 minutes, with 10 minutes resting period between each two cigarettes. The animals were exposed initially to one cigarette per day, gradually increasing to a maximum of 5 cigarettes per day over a 20 days period and continued for 70 days. Therefore the total exposure period to cigarette smoke was 3 consecutive months.

### Animals and groups

Thirty six guinea pigs (600–800 g) were randomly divided to 6 groups as follows

1. Control group: animals were exposed to ambient air (n = 6).
2. COPD group: animals were exposed to cigarette smoke (n = 9).
3. COPD+intratracheal PBS (COPD-ITPBS): animals were exposed to cigarette smoke and then received intratracheal PBS as vehicle (n = 5).
4. COPD+intratracheal ASCs (COPD-ITASC): animals were exposed to cigarette smoke and then received 10^6^ intratracheal ASCs (n = 6).
5. COPD+intravenous PBS (COPD-IVPBS): animals were exposed to cigarette smoke and then received intravenous PBS as vehicle (n = 5).
6. COPD+intravenous ASCs (COPD-IVASC): animals were exposed to cigarette smoke and then received 10^6^ intravenous ASCs (n = 5).

The sample sizes of the groups were chosen according our previous study [14]. At the beginning of the study, for control groups 6 animals and for other group 9 animals were chosen but, in groups 3, 5 and 6, 4 animals and in group 4, 3 guinea pigs were died which was not due to cigarette smoke exposure because in non treated exposed group there was not any animal death. All animals were kept in a temperature controlled room with access to standard food and water *ad libitum* and were maintained at 22±2°C on a 12 h light/dark cycle. Experiments were performed in compliance with the rulings of the Institute of Laboratory Animals Resources, Commission on Life Sciences [15]. The study was approved by the Ethical Committee of Mashhad University of Medical Sciences. All measurements were done 14 days after treatment with cell or vehicle.

### Preparation and administration of stromal cells

Healthy guinea pigs with weight range of 600 to 800 g were anesthetized by intraperitoneal injection of ketamine (150 mg/kg) and xylazine (6 mg/kg). Subcutaneous inguinal fat pads were removed and minced into 1–2 mm pieces. The tissue pieces were incubated at 37°C for 60 minutes in PBS containing 2 mg/ml collagenase while being shaken (60 cycles/min). After centrifugation (2000 rpm for 5 minutes), the floated lipid layer was removed and the stroma-vascular fraction was collected, washed and re-suspended in DMEM medium supplemented with 10% FBS, 100 units/ml penicillin and 100 µg/ml streptomycin [16], [17]. The cells were seeded into tissue culture flask and passaged after they were grown to 60–80% confluency; they were used at passages three to six.

For administration of ASCs, the guinea pigs were anesthetized. In COPD-ITASC group, the trachea and in COPD-IVASC group, the jugular vein was exposed and 0.3 ml PBS containing 10^6^ cells was injected using a 27 gauge insulin syringe. Only 0.3 ml PBS was administered to COPD-ITPBS and COPD-IVPBS groups. Viability of injected cells was found to be more than 90% as assessed with trypan blue staining.

### Differentiation of stromal cell

To test differentiating ability of stromal cells to adipocyte, they were seeded in 12-well culture plate and then incubated in DMEM supplemented with 3% FBS, 66 µM biotin, 250 µM IBMX, 1 µM dexamethasone, 34 µM d-panthothenate, 5 µM indomethacin and 0.2 µM insulin. The cells were maintained in differentiation medium for 3 days and then exposed to the adipocyte maintenance medium consisting of DMEM supplemented with 3% FBS, 66 µM biotin, 1 µM dexamethasone, 34 µM d-panthothenate and 0.2 µM insulin. After additional 9 days of incubation, adipogenesis was confirmed so that the cells were fixed with 10% formalin, incubated with Oil Red O solution, washed three times with distilled water and photographed using inverted microscope [18].

For osteocyte differentiation, the stromal cells were incubated in DMEM supplemented with 10% FBS, 10 µg/ml ascorbic acid, 5 mM β-glycerol phosphate and 0.1 µM dexamethasone. The cells were maintained in the differentiation medium for two weeks and the culture medium was replaced every 3 days [19]. For Alizarin red staining, the cells were fixed with 10% formalin, incubated with 2% Alizarin red solution, washed three times with distilled water and photographed using inverted microscope [20].

### Stromal cell labeling and tracing

The cells were suspended in PBS and incubated with 2 µM cell tracker CM-DiI (Invitrogen) for 5 min in 37°C and then 15 min in 4°C. Then, the cells were centrifuged for two times, suspended in 0.3 ml PBS and prepared for injection to the animal. After either 2 days or 2 weeks of ASCs administration (either intratracheal or intravenous) the animals were euthanized and 4 µm sections were provided from different regions of the lung. Existence of the labeled cells was detected under fluorescent microscope.

### Measurement of tracheal responsiveness

The animals were sacrificed by a blow on its neck, and their tracheal were removed. Then each trachea was cut into 10 rings (each containing 2–3 cartilaginous rings), all the rings were then cut open opposite the trachealis muscle, and sutured together to form a tracheal chain [21]. The tissue was then suspended in a 20 ml organ bath (Schuler organ bath type 809, March-Hugstetten, Germany) containing Krebs-Henseliet solution of the previously described composition [22]. The Krebs solution was maintained at 37°C and gassed with 95% O_2_ and 5% CO_2_. Tissue was suspended under isotonic tension of 1 g and allowed to equilibrate for at least 1 h while being washed with Krebs solution every 15 min. Responses were measured using vernier control type 850 N sensor with sensitivity range: 0–20 g and resolution: 0.2 mm/turn (Hugo-Sachs Elektronik, Germany), amplified with amplifier (ML/118 quadribridge amp, March-Hugstetten, Germany) and recorded on powerlab (ML-750, 4 channel recorder, March-Hugstetten, Germany).

Consecutive concentrations of methacholine hydrochloride (Sigma Chemical Ltd, U.K.) which induce tracheal chain contraction (including 10^−7^ to 10^−5^ mM, dissolved in saline) were added every 2 min. The contraction due to each concentration was recorded at the end of each 2 min, which the effect reached a plateau in all experiments. To obtain cumulative concentration-response curve, percentage of tracheal smooth muscle contraction in proportion to maximum response obtained by its final concentration was plotted against log concentration of methacholine. The effective concentration of methacholine, causing 50% of maximum response (EC_50_) was measured from methacholine response curve in each experiment.

### Total and differential WBC count in BALF

After sacrificing the animals, broncho-alveolar lavage fluid (BALF) was prepared from the lung by locating a cannula into trachea and lavaging the lungs with 2 ml of saline for 5 times (total: 10 ml). One ml of BALF was stained with Turk solution and counted in duplicate in a hemocytometer (in a Burker chamber). For differential WBC count, BALF was centrifuged at 2500*×g* at 4°C for 10 min. The smear was prepared from the cells and stained with Wright-Giemsa. Differential cell analysis was carried out under a light microscope by counting 100 cells and calculating percentage of each cell type.

### Cytokine measurements

For measurements of interleukin-8 (IL-8) in BALF, the fluid was centrifuged at 2500*×g* for 10 min and the supernatant was collected for measurement of interleukin-8 (IL-8) concentration. Five ml blood sample was also collected from the left ventricle and placed in a citrate containing blood collection tube. The blood was centrifuged and the serum was separated and kept in −70°C for further measurement of IL-8 concentrations. The concentration of this parameter was assayed using a double antibody sandwich enzyme-linked immunosorbent assay kit (HANGZHOU EASTBIOPHARM CO., LTD., Hangzhou) according to the manufacturer’s protocol.

### Statistical analysis

All data are expressed as mean ± SEM and analyzed by Instat software. Comparison of data was performed using unpaired “t” test and Analysis of Variance with Tukey-Cramer post test. Significance was accepted at p<0.05.

## Results

### Characterization of stromal cell

**Figure 1:**
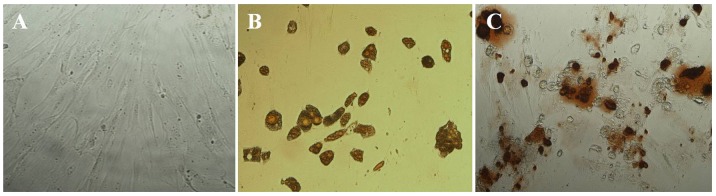
Differentiation of adipose stromal cells to adipocyte and osteocyte lineage. The stromal cells were cultured in adipogenic or osteogenic differentiating media for 12 and 14 days, respectively. (A) Stromal cells cultured in control media; (B) Oil Red O staining of cultured cells in adipogenic differentiating media; (C) Alizarin Red staining of cells cultured in osteogenic differentiating media. Magnification ×100.

Morphology of stromal cells isolated from adipose tissue of guinea pig showed that they are adherent and have significant expansion in the cultures (Fig. 1A). After passage 3, they had spindled fibroblast-like appearance that is consistent with MSCs morphology. Staining the differentiated adipocytes with Oil Red O showed accumulated triglyceride droplets in the cell cytoplasm which confirms adipogenic differentiation capacity of the isolated stromal cells (Fig. 1B). In addition, Alizarin Red staining of the cells cultured in differentiating media specific for osteocyte revealed extracellular matrix mineralization which confirms osteogenic differentiation capacity of the stromal cells (Fig. 1C).

### Stromal cell detection in the lung

**Figure 2:**
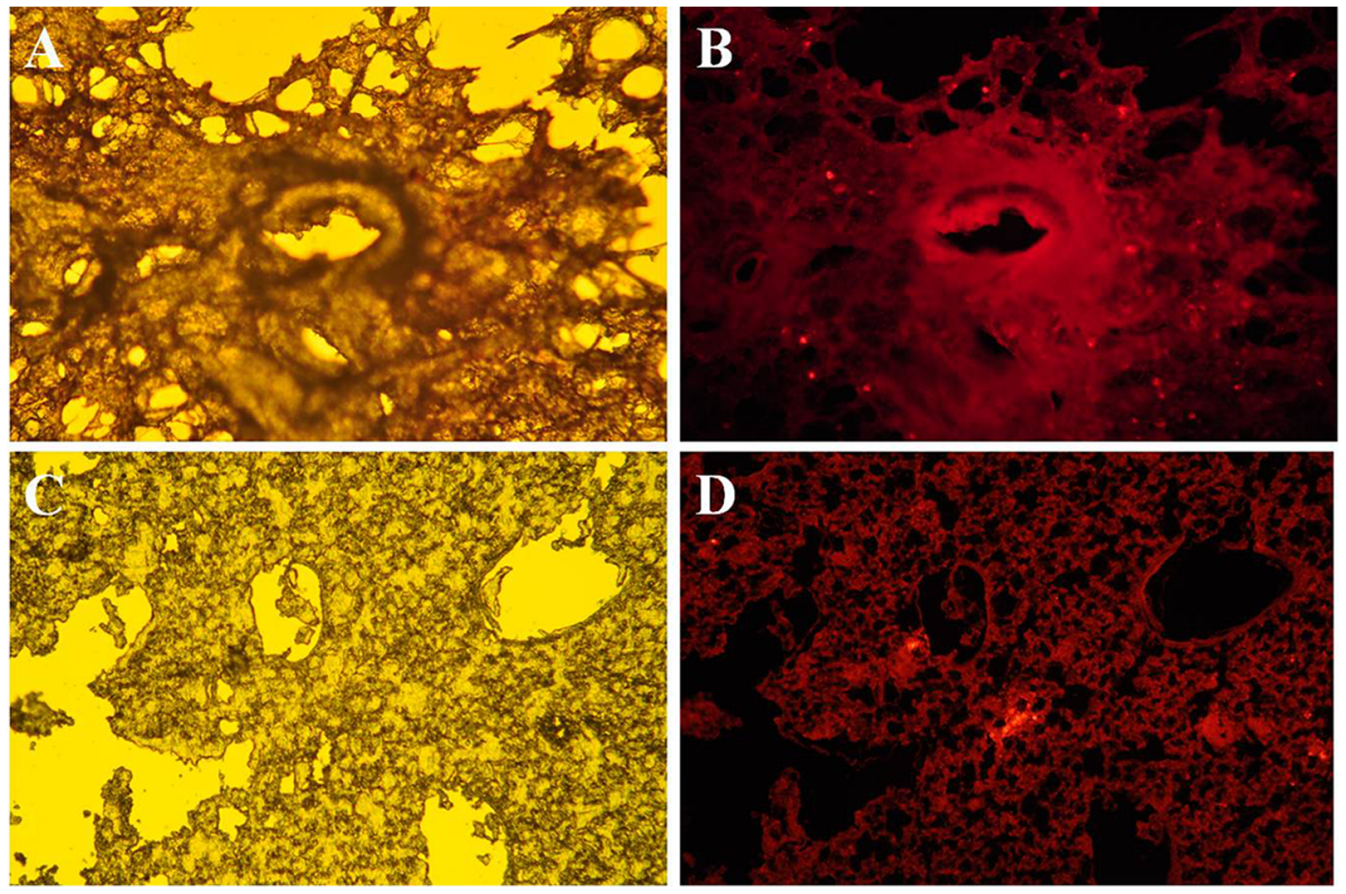
Fluorescence microscopic photographs of lungs after intratracheal or systemic delivery of CM-DiI-labeled stromal cells. (A) Image of lung from COPD animal harvested 14 days after injection of labeled cells into trachea. Magnification ×100; (B) Image of lung from COPD animal harvested 14 days after injection of labeled cells into jugular vein, Magnification ×400.

The CM-DiI-labeled stromal cells were detected in the lung of COPD guinea pigs 14 days after intratracheal administration (Fig. 2A). Fluorescence microscopy of lung sections either 2 or 14 days after intravenous cell injection also showed the labeled cells in the lung alongside resident cells in airways and alveolar structures (Fig. 2B).

### Tracheal responsiveness

**Figure 3:**
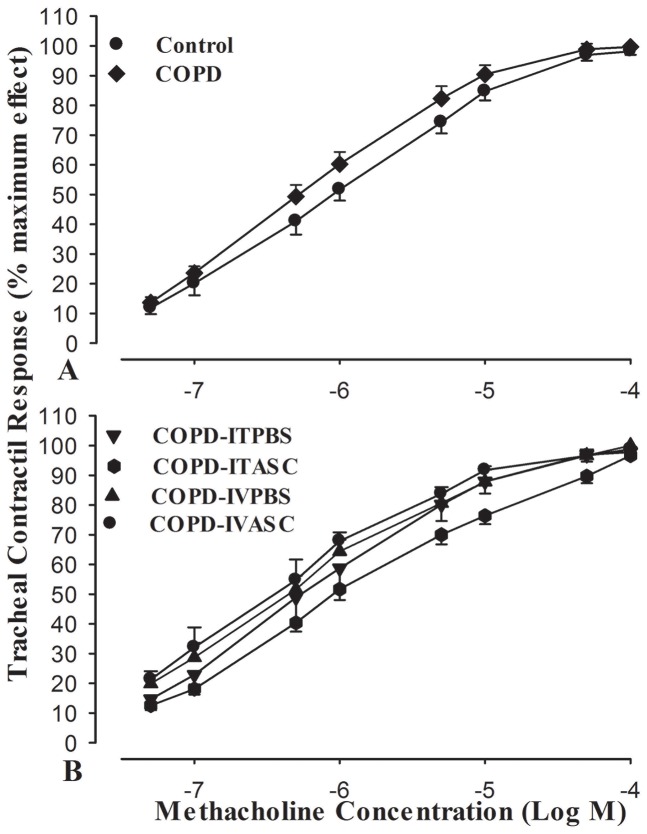
Cumulative concentration-response curves of methacholine induced trachea contraction. (A) Control and cigarette smoke exposed animals (an animal model of COPD); (B) COPD-ITPBS, COPD-ITASC, COPD-IVPBS and COPD-IVASC groups. The animals in COPD-ITPBS, COPD-ITASC, COPD-IVPBS and COPD-IVASC groups were exposed to cigarette smoke for 3 months and then received intratracheal or intravenous injection of PBS or ASCs.

**Figure 4:**
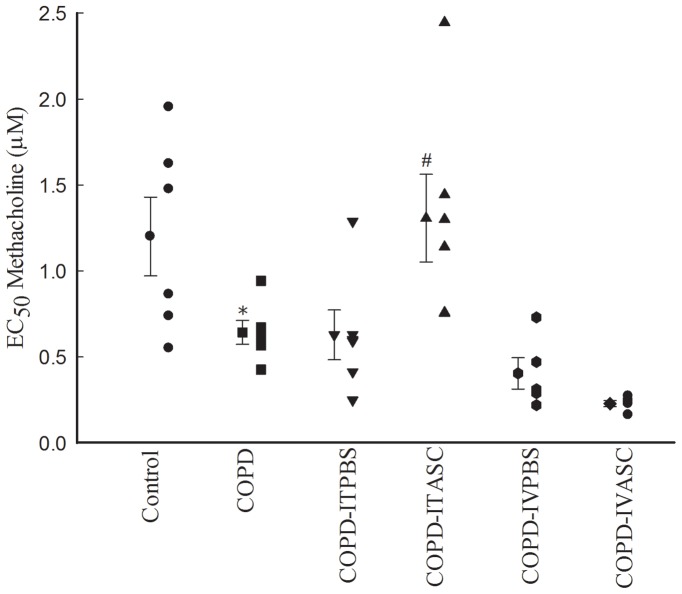
Effect of adipose derived stromal cells (ASCs) therapy on EC_50_ methacholine (the effective concentration caused 50% of maximum contraction response to methacholine) in different groups of animals. Data are presented as mean ± SEM (big symbols with bars). The animals in COPD-ITPBS, COPD-ITASC, COPD-IVPBS and COPD-IVASC groups were exposed to cigarette smoke for 3 months and then received intratracheal or intravenous injection of PBS or ASCs. *P<0.05 as compared with control group. ^#^ *P*<0.05 as compared with COPD-ITPBS group.

Methacholine concentration-response curves of guinea pigs exposed to cigarette smoke showed left ward shift compared to those of control animals (Fig. 3A, Table S1). However, the curves in COPD-ITASC showed right ward shift compared to COPD- ITPBS (Fig. 3B, Table S1). In COPD animals, EC_50_ of methacholine was significantly lower compared to that of control group (*P*<0.05). There was no significant difference between EC_50_ of the COPD, COPD-ITPBS and COPD-IVPBS groups (Fig. 4, Table S2). In COPD-ITASC group, EC_50_ significantly increased (*P*<0.05) compared to COPD-ITPBS while no significant change was observed in EC_50_ of COPD-IVASC group compared to the COPD-IVPBS animals (Fig. 4, Table S2).

### Concentration of IL-8 in serum and BALF

**Figure 5:**
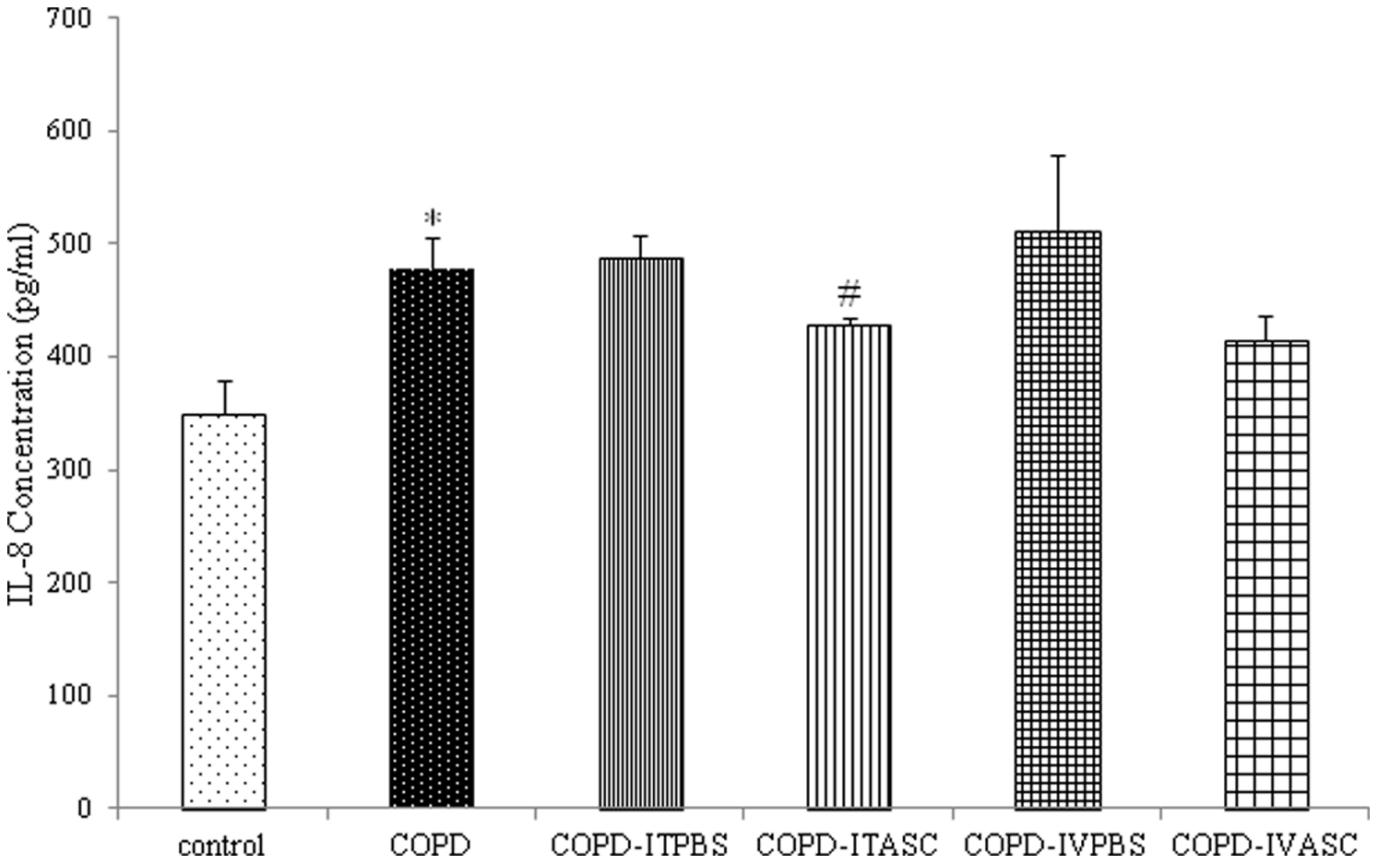
Serum level of IL-8 in different groups of animals. The animals in COPD-ITPBS, COPD-ITASC, COPD-IVPBS and COPD-IVASC groups were exposed to cigarette smoke for 3 months and then received intratracheal or intravenous injection of PBS or ASCs. Data are shown as mean± SEM. *P<0.05 as compared with control group. ^#^ *P*<0.05 as compared with COPD-ITPBS group.

**Figure 6:**
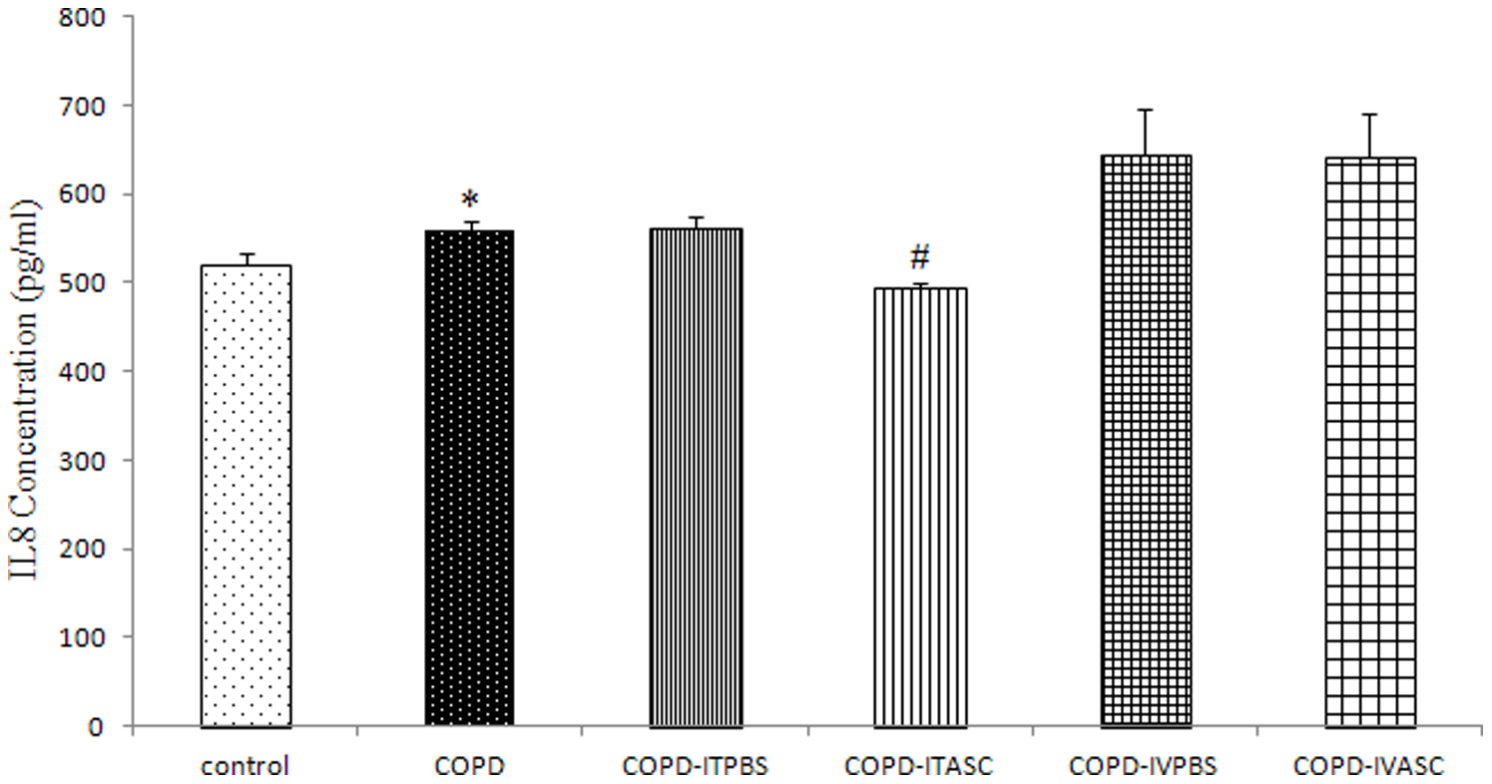
Concentration of IL-8 in BALF of different groups of animals. The animals in COPD-ITPBS, COPD-ITASC, COPD-IVPBS and COPD-IVASC groups were exposed to cigarette smoke for 3 months and then received intratracheal or intravenous injection of PBS or ASCs. Data are shown as mean± SEM. *P<0.05 as compared with control group. ^#^ *P*<0.05 as compared with COPD-ITPBS group.

The level of IL-8 in serum and BALF in COPD animals was significantly higher compared to control (*P*<0.05 for both cases). However, concentration of IL-8 was not significantly different between COPD, COPD-ITPBS and COPD-IVPBS groups both in serum and BALF (Fig. 5 and 6, Table S3 and S4). A decline in IL-8 concentration was observed in both serum and BALF (*P*<0.05 for both cases) of COPD-ITASC animals compared to the COPD-ITPBS group (Fig. 5 and 6, Table S3 and S4).

### Total and differential WBC count in BALF

**Figure 7:**
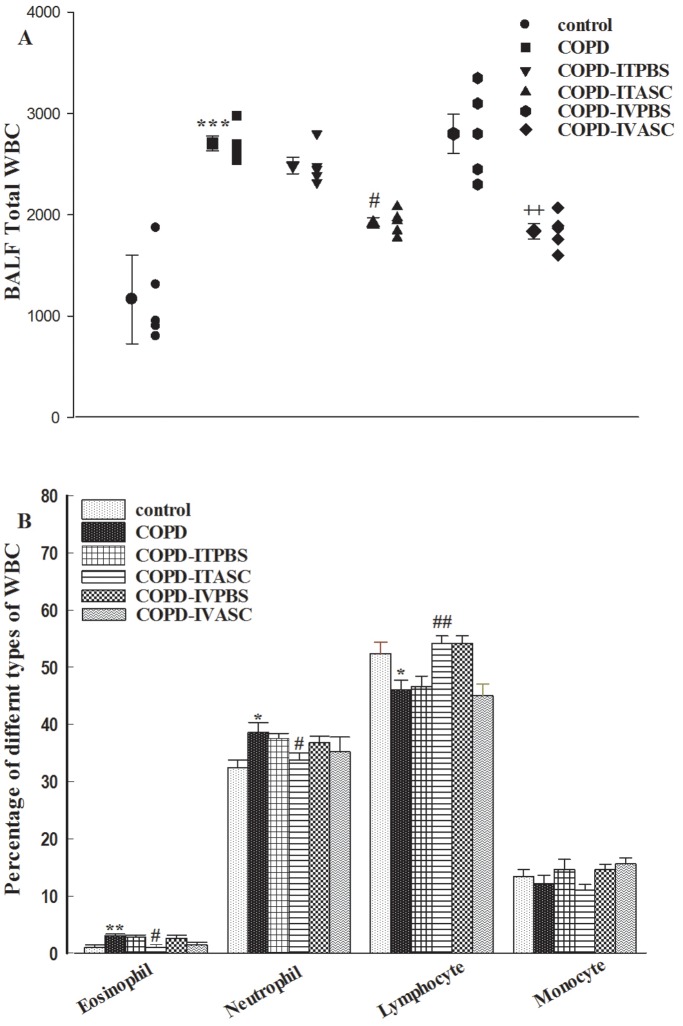
Total (A) and differential (B) WBC counts in BALF of different groups of animals. The animals in COPD-ITPBS, COPD-ITASC, COPD-IVPBS and COPD-IVASC groups were exposed to cigarette smoke for 3 months and then received intratracheal or intravenous injection of PBS or ASCs. Data are shown as mean± SEM. *P<0.05, **P<0.01 and ***P<0.001 as compared with control group. ^#^ *P*<0.05 and ^##^ *P*<0.01 as compared with COPD-ITPBS group. ^++^ *P*<0.01 as compared with COPD-IVPBS group.

Total WBC in the COPD was significantly higher compared to the control group (*P*<0.001). No significant difference was observed between COPD-ITPBS, COPD-IVPBS and COPD groups (Fig. 7A, Table S5). Cell therapy could decrease this parameter in BALF of both COPD-ITASC and COPD-IVASC compared to their corresponding PBS groups (*P*<0.05 and *P*<0.01, respectively).

The percentage of eosinophils and neutrophils increased but lymphocytes decreased significantly in BALF of COPD compared to control group (*P*<0.05 to *P*<0.01). The changes in all differential WBC counts were significantly reversed in COPD-ITASC compared to COPD-ITPBS group (p<0.05 to p<0.01). However, there was no significant difference in differential WBC counts between COPD-IVASC compared to COPD-IVPBS group (Fig. 7B, Table S6).

## Discussion

In the present study, guinea pigs were exposed to cigarette smoke to induce an animal model of COPD with physiological and pathological changes such as increased airway responsiveness, airflow obstruction and pulmonary inflammation. It has been shown that long-term cigarette smoke develops phenotype of COPD such as airflow obstruction and emphysema [23], [24], tracheal hyperresponsiveness and lung inflammation [4] which are main pathophysiological features of COPD. A considerable destruction of alveolar walls and widespread emphysema in H&E stained lung sections of cigarette exposed guinea pigs were also shown in our previous study using similar method of exposing guinea pigs to cigarette smoke [25]. Therefore, the results of our present and previous [25] studies confirm the induction of COPD in the animals. Although bronchial hyperresponsiveness is the main characteristic feature of asthma, it is also well documented in animals exposed to cigarette smoke [14], [26]–[29], smokers [30] and COPD patients [31].

The COPD animals were treated with intra-tracheal or intra-jugular stromal cells. The labeled stromal cells were detectable in the lung even 2 weeks after cell therapy. Therefore, a two weeks recovery is a proper time for the stromal cells to exert their impact by either regeneration in the damaged tissue or paracrine effects which is supported by previous studies [32].

In the present study, tracheal responsiveness to methacholine in COPD group significantly increased compared to control group which is supported by findings of previous studies in rats [4], guinea pigs [27] and mice [33]. There was no significant difference in tracheal responsiveness between the COPD-ITPBS, COPD-IVPBS and COPD group indicating PBS had no effect on tracheal responsiveness. Tracheal response in animals treated with intratracheal ASCs decreased (EC_50_ increased) which may be due to the paracrine effects of stromal cells in the bronchus [34].

The mechanism of increased bronchial smooth muscle contraction due to the cigarette smoke is suggested to be involvement of an augmented agonist-induced Ca^2+^ sensitization [3], airway epithelial injury and mucosal inflammation [26] as well as the participation of tachykinins released from afferent nerve fibers innervating the airways [26], [35]. There is evidence supporting the idea that cholinergic tone of the airways is increased in COPD patients [3]. The mechanisms of beneficial action of ASCs on tracheal responsiveness are yet to be elucidated. Schweitzer et al. reported that ameliorative properties of ASCs on lung injury are attributed to secretion of different factors which have paracrine effects on lung and systemic injury [8]. Katsha et al. suggested that intratracheal administration of MSCs isolated from ASCs ameliorate lung injury through humoral factors which up-regulate growth factors and reduce proinflammatory cytokines (e.g. IL-1β) [10]. Hence, the paracrine effects of these cells injected directly to the point of damage may justify their protective effects. However, in our study no significant change was observed in COPD-IVASC which might be explained by the limited access of systemically delivered cells to the trachea and bronchi. Another reason of the observed result might be due to the inevitable low number of injected cells. In this work, the number of stromal cells was intentionally chosen to be low because it was reported that intravenous injection of high number of MSC may lead to embolism in the lung [35].

Inflammatory cells that play key roles in COPD include macrophages, neutrophils and T-lymphocytes. Several inflammatory mediators contributing to disease development are tumor necrosis factor (TNF)-α, IL-8, monocyte chemoattractant protein-1 and proteases such as matrix metalloproteinases [36]. Our data showed that 3 months of cigarette smoke exposure in COPD, COPD-ITPBS and COPD-IVPBS groups significantly increased lung inflammation as indicated by elevated number of inflammatory cells (total WBC) in BALF and inflammatory mediator, IL-8, in both serum and BALF. Total and differential WBC in BALF as well as the levels of IL-8 in both serum and BALF were decreased in COPD-ITASC animals. Anti-inflammatory effects of mesenchymal stem cells have been previously proven and support the results of the present study [12]. The effect of adipose stem cell on cigarette smoke-induced lung inflammation [8] and effect of MSCs on bleomycin-induced inflammation in lung [37] were also shown which are consistent with the findings of the present study.

Although, the sample sizes of the groups were chosen according our previous study [14], in groups 3, 5 and 6, four animals and in group 4, three guinea pigs were died during surgery or postoperative days. This mortality was not due to cigarette smoke exposure because in non-treated exposed group there was not any animal death. In addition, cell therapy side effects (e.g. infection or embolism) per se cannot be responsible for such mortality since a comparable rate of death was seen also in COPD-ITPBS and COPD-IVPBS groups. We guess that our surgical procedures (exposing trachea and jugular vein under deep anesthesia) have been invasive for guinea pigs model of COPD which suffer from obstructive bronchiolitis, airway hyperresponsiveness, emphysema and lung inflammation (2–4,23,24). Thus, for future works, we suggest harmless routes for cell delivery such as using tracheal tube intubation and peripheral vein injection for intratracheal and intravenous administration, respectively.

## Conclusion

In conclusion, tracheal responsiveness, level of IL-8 in both serum and BALF as well as total and differential WBC of BALF which are induced by cigarette smoke exposure can more feasibly be decreased by intratracheal ASC injection which indicates a promising therapeutic effect for COPD.

## Supporting Information

Table S1 **Tracheal smooth muscle contraction due to cumulative concentration of methacholine.**
(DOCX)

Table S2 **EC50 values.**
(DOCX)

Table S3 **Serum level of IL-8.**
(DOCX)

Table S4 **BALF level of IL-8.**
(DOCX)

Table S5 **BALF total WBC.**
(DOCX)

Table S6 **BALF differential WBC.** N: neutrophil, L: lymphocyte, M: monocyte, E: eosinophil.
(DOCX)
