# Expression of Concern: Adipose-derived stromal cell therapy affects lung inflammation and tracheal responsiveness in Guinea Pig model of COPD

**DOI:** 10.1371/journal.pone.0328264

**Published:** 2025-07-16

**Authors:** The PLOS One Editors

Following the publication of this article [1] concerns were raised with the results presented in the article, specifically:

- Figs 1B, 1C, 2C, and 2D presented in this article [1] were previously reported in Figs 1B, 1D, 3C, and 3D of [2] respectively.
- The statistical approach reported in this article is inappropriate for the described study.

**Fig 1:**
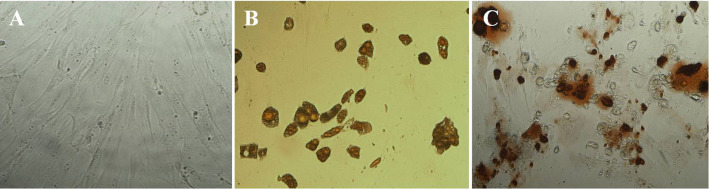
Differentiation of adipose stromal cells to adipocyte and osteocyte lineage. The stromal cells were cultured in adipogenic or osteogenic differentiating media for 12 and 14 days, respectively. (A) Stromal cells cultured in control media; (B) Oil Red O staining of cultured cells in adipogenic differentiating media; (C) Alizarin Red staining of cells cultured in osteogenic differentiating media. Magnification ×100. Figure panels 1B and 1C were previously published in [2].

**Fig 2:**
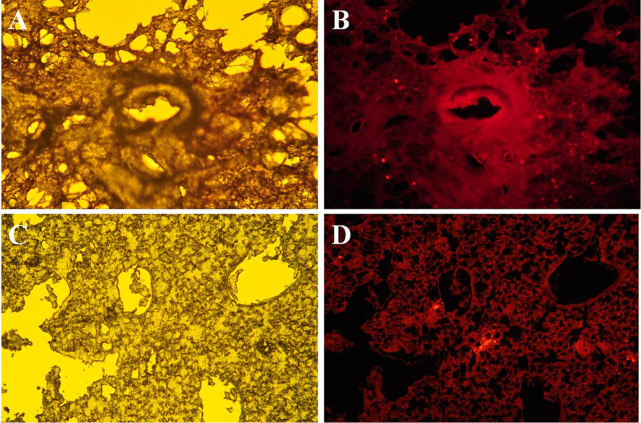
Microscopic photographs of lungs after intratracheal or systemic delivery of CM-DiI-labeled stromal cells. (A) Image of lung from COPD animal harvested 14 days after injection of labeled cells into trachea (magnification ×100). (B) fluorescent photomicrograph from the same field of view as image A. (C) Image of lung from COPD animal harvested 14 days after injection of labeled cells into jugular vein (magnification ×100). (D) fluorescent photomicrograph from the same field of view as image C. Figure panels 2C and 2D were previously published in [2].

The authors commented that Figs 1B, 1C, 2C, and 2D of this article [1] and Figs 1B, 1D, 3C, and 3D of [2] were intentionally reused as they represent results from a preliminary study carried out by the same research group. The figure legend of Figs 1 and 2 are updated to reflect the previous reporting of these results. Figs 1B, 1C, 2C, and 2D in [1] were previously published under an Open Access CC BY 2.0 license [3]. Please provide due attribution to the original publication [2] when referring to this content.

In addition, the figure legend for Fig 2 is updated to correct errors involving incomplete and inaccurate labeling of the published panels. The corresponding paragraph in the Results section under the sub header “**Stromal cell detection in the lung**” should read:

“The CM-DiI-labeled stromal cells were detected in the lung of COPD guinea pigs 14 days after intratracheal administration (Fig. 2A and 2B). Fluorescence microscopy of lung sections 14 days after intravenous cell injection also showed the labeled cells in the lung alongside resident cells in airways and alveolar structures (Fig. 2C and 2D).”

The methodology section of the article reports that “*Comparison of data was performed using unpaired “t” test and Analysis of Variance with Tukey-Cramer post test.*”. This statistical approach was assessed by an independent statistical reviewer who concluded that the reported sample sizes are relatively small, and it is unclear whether the responses are distributed as Gaussian. The statistical expert suggested that nonparametric tests, such as Wilcoxon rank sum, and Kruskal Wallis tests, followed by FDR-adjusted p-values for multiple comparisons would have been more appropriate.

The authors did not respond to the statistical concerns raised or the journal’s request that they reanalyze their data in line with the statistical recommendations received by the journal. The raw data underlying the results presented in Figs 4-7Figure 5Figure 6Figure 7 remain available in the Supporting Information files of [1]. However, in the absence of a statistical reanalysis it remains unclear whether the reported results and conclusions are supported by the data.

The *PLOS One* Editors issue this Expression of Concern to inform readers to interpret the findings of this article with caution in light of the unresolved statistical concerns. With the information in this notice, PLOS considers the errors affecting Fig 2 resolved.
