# Supplementary material for: Adipose-Derived Stromal Cell Therapy Affects Lung Inflammation and Tracheal Responsiveness in Guinea Pig Model of COPD
Source: PLoS One. 2014 Oct 20;9(10):e108974. doi: 10.1371/journal.pone.0108974 (PMC4203716; doi:10.1371/journal.pone.0108974)
Supplement: Table S1 — Tracheal smooth muscle contraction due to cumulative concentration of methacholine. (DOCX) [file pone.0108974.s001.docx]

Table Supplement 1- Tracheal smooth muscle contraction due to cumulative concentration of methacholine

| Methacholine Concentration (µM) | Control | COPD | COPD-ITPBS | COPD-ITASC | COPD-IVPBS | COPD-IVASC |
| --- | --- | --- | --- | --- | --- | --- |
| 0.05  0.1  0.5  1  5  10  50  100  500  1000 | 11.90±2.10  20.11±4.03  40.98±4.45  51.66±3.69  74.26±3.69  84.68±2.30  96.94±1.92  98.17±1.19  100.00±0.00  100.00±0.00 | 13.70±1.80  23.60±2.24  49.30±3.91  60.22±4.11  82.30±4.11  90.42±3.11  98.87±1.83  99.65±0.69  100.00±0.00  100.00±0.00 | 14.70±2.20  22.90±3.24  48.90±8.12  58.70±7.97  80.10±5.51  87.90±4.04  96.90±2.34  97.80±1.12  100.00±0.20  100.00±0.00 | 12.60±1.60  18.08±1.86  40.40±2.98  51.70±3.67  69.98±3.24  76.37±2.81  89.65±2.34  96.63±0.89  100.00±0.00  100.00±0.00 | 9.90±4.20  28.74±10.10  51.60±10.05  64.40±6.41  80.60±5.38  87.80±4.13  96.60±2.09  100.00±0.00  100.00±0.00  100.00±0.00 | 21.30±1.40  32.10±1.52  54.70±1.50  67.90±0.44  83.80±0.91  91.70±1.31  96.56±1.54  98.73±0.82  99.90±0.10  100.00±0.00 |
