# Supplementary material for: Adipose-Derived Stromal Cell Therapy Affects Lung Inflammation and Tracheal Responsiveness in Guinea Pig Model of COPD
Source: PLoS One. 2014 Oct 20;9(10):e108974. doi: 10.1371/journal.pone.0108974 (PMC4203716; doi:10.1371/journal.pone.0108974)
Supplement: Table S2 — EC50 values. (DOCX) [file pone.0108974.s002.docx]

Table Supplement 2- EC50 values.

| No | Control | COPD | COPD-ITPBS | COPD-ITASC | COPD-IVPBS | COPD-IVASC |
| --- | --- | --- | --- | --- | --- | --- |
| 1  2  3  4  5  6 | 1.95  0.74  1.62  1.48  0.86  0.55 | 0.94  0.67  0.43  0.57  0.60  0.65 | 1.29  0.25  0.60  0.41  0.59  0.63 | 2.45  1.14  0.75  0.76  1.30  1.44 | 0.73  0.22  0.31  0.47  0.29 | 0.25  0.16  0.27  0.23  0.23 |
