# Supplementary material for: Adipose-Derived Stromal Cell Therapy Affects Lung Inflammation and Tracheal Responsiveness in Guinea Pig Model of COPD
Source: PLoS One. 2014 Oct 20;9(10):e108974. doi: 10.1371/journal.pone.0108974 (PMC4203716; doi:10.1371/journal.pone.0108974)
Supplement: Table S3 — Serum level of IL-8. (DOCX) [file pone.0108974.s003.docx]

Table Supplement 3- Serum level of IL-8.

| No | Control | COPD | COPD-ITPBS | COPD-ITASC | COPD-IVPBS | COPD-IVASC |
| --- | --- | --- | --- | --- | --- | --- |
| 1  2  3  4  5  6 | 435.53  243.48  330.14  431.63  351.99  304.37 | 480.82  351.22  527.66  490.97  505.80  519.07 | 508.14  547.18  439.44  506.58  437.88  487.84 | 448.81  422.26  432.41  419.92  403.53  444.12 | 668.00  523.00  368.00  347.00  648.00 | 416.02  483.94  415.24  348.09  415.00 |
