# Supplementary material for: Adipose-Derived Stromal Cell Therapy Affects Lung Inflammation and Tracheal Responsiveness in Guinea Pig Model of COPD
Source: PLoS One. 2014 Oct 20;9(10):e108974. doi: 10.1371/journal.pone.0108974 (PMC4203716; doi:10.1371/journal.pone.0108974)
Supplement: Table S4 — BALF level of IL-8. (DOCX) [file pone.0108974.s004.docx]

Table Supplement 4- BALF level of IL-8.

| No | Control | COPD | COPD-ITPBS | COPD-ITASC | COPD-IVPBS | COPD-IVASC |
| --- | --- | --- | --- | --- | --- | --- |
| 1  2  3  4  5  6 | 494.87070  521.41552  547.17961  488.00000  551.86399  520.66596 | 561.23275  542.49523  539.37231  583.87392  588.55830  550.30253 | 551.86399  608.07655  557.03012  543.27596  560.00000  564.04932 | 487.06340  490.96705  488.62486  516.73114  490.00000  494.67729 | 551.2  700.0  796.0  668.0  511.0 | 601.04998  552.64472  836.04971  578.40881  640.00000 |
