# Supplementary material for: Adipose-Derived Stromal Cell Therapy Affects Lung Inflammation and Tracheal Responsiveness in Guinea Pig Model of COPD
Source: PLoS One. 2014 Oct 20;9(10):e108974. doi: 10.1371/journal.pone.0108974 (PMC4203716; doi:10.1371/journal.pone.0108974)
Supplement: Table S5 — BALF total WBC. (DOCX) [file pone.0108974.s005.docx]

Table Supplement 5- BALF total WBC.

| No | Control | COPD | COPD-ITPBS | COPD-ITASC | COPD-IVPBS | COPD-IVASC |
| --- | --- | --- | --- | --- | --- | --- |
| 1  2  3  4  5  6 | 950  1870  1310  800  900  1166 | 2680  2700  2540  2630  2980  2706 | 2390  2450  2800  2320  2480  2488 | 1770  1940  2080  1970  1840  1920 | 2800  2450  3100  2300  3350 | 2070  1890  1600  1870  1760 |
