# Supplementary material for: Adipose-Derived Stromal Cell Therapy Affects Lung Inflammation and Tracheal Responsiveness in Guinea Pig Model of COPD
Source: PLoS One. 2014 Oct 20;9(10):e108974. doi: 10.1371/journal.pone.0108974 (PMC4203716; doi:10.1371/journal.pone.0108974)
Supplement: Table S6 — BALF differential WBC. N: neutrophil, L: lymphocyte, M: monocyte, E: eosinophil. (DOCX) [file pone.0108974.s006.docx]

Table Supplement 6- BALF differential WBC.

| Dif | No | Control | COPD | COPD-ITPBS | COPD-ITASC | COPD-IVPBS | COPD-IVASC |
| --- | --- | --- | --- | --- | --- | --- | --- |
| N | 1  2  3  4  5  6 | 36  30  29  32  35  33 | 44  36  34  40  39  39 | 38  35  37  40  38  39 | 35  29  34  36  35  34 | 35  33  42  39  27 | 35  37  35  41  36 |
| L | 1  2  3  4  5  6 | 49  58  48  56  51  52 | 42  47  42  50  49  45 | 40  49  46  47  51  45 | 54  58  56  52  51  53 | 52  47  42  41  43 | 46  47  47  41  49 |
| M | 1  2  3  4  5  6 | 15  12  17  10  13  13 | 9  12  17  13  10  11 | 20  12  17  10  14  15 | 10  11  8  12  14  12 | 12  18  15  17  16 | 15  13  16  17  12 |
| E | 1  2  3  4  5  6 | 0  0  2  2  1  1 | 4  3  2  3  3  3 | 2  4  2  3  3  3 | 1  2  2  0  0  1 | 1  2  1  3  0 | 4  3  2  1  3 |

N: neutrophil, L: lymphocyte, M: monocyte, E: eosinophil
